# Perceptual integration and attention in human extrastriate cortex

**DOI:** 10.1038/s41598-017-13921-z

**Published:** 2017-11-01

**Authors:** Francesca Strappini, Gaspare Galati, Marialuisa Martelli, Enrico Di Pace, Sabrina Pitzalis

**Affiliations:** 1 Neurobiology Department, Weizmann Institute of Science, Rehovot, Israel; 2 Department of Psychology, Sapienza University, Rome, Italy; 3 Neuropsychology Center, Santa Lucia Foundation, Rome, Italy; 4 Department of Education in Sport and Human Movement, University of Rome “Foro Italico”, Rome, Italy

## Abstract

Visual crowding is a perceptual phenomenon with far-reaching implications in both perceptual (e.g., object recognition and reading) and clinical (e.g., developmental dyslexia and visual agnosia) domains. Here, we combined event-related fMRI measurements and wide-field brain mapping methods to investigate whether the BOLD response evoked by visual crowding is modulated by different attentional conditions. Participants underwent two sessions of psychophysical training outside the scanner, and then fMRI BOLD activity was measured simultaneously in early visual areas (including the visual word form area, VWFA), while they viewed strongly-crowded and weakly-crowded Gabor patches in attended and unattended conditions. We found that crowding increased BOLD activity in a network of areas including V1, V2, V3A, V4/V8, and VWFA. In V4/V8 and VWFA we found an increased activity related to attention. The effect of crowding in V1 was recorded only when attention was fully devoted to the target location. Our results provide evidence that some area beyond V1 might be the likely candidate for the site of crowding, thus supporting the view of visual crowding as a mid-level visual phenomenon.

## Introduction

Most of the time, objects fall in the peripheral portion of our visual field and are not recognizable because they are cluttered with the nearby objects. For recognition to succeed, we move our eyes around the scene and look at the objects through the central portion of the retina, where the clutter is less damaging. Crowding refers to the failure to identify an object when surrounded by closely spaced elements^1,2^. It is a well-studied psychophysical phenomenon that characterizes foveal strabismic amblyopic vision and normal peripheral recognition^3–5^. It also affects foveal vision when objects are closely spaced and have a small size of few minutes of arc (i.e., only slightly above acuity^6,7^).

Crowding is a mid-level visual phenomenon sparing detection and wrecking recognition, whereby correctly detected features are integrated over a region of the visual field too large to isolate the target object from the surrounding elements^8–10^. Crowding is an operationally defined phenomenon with models that attempt to provide an account for the computation that occurs within the integration region^8,11–15^.

Critical spacing (or Bouma’s window)^16^, the center-to-center spacing between the target object and the surrounding elements needed for recognition, measures the size of the integration region whose size increases with eccentricity^16^. Specifically, we know that task and stimuli for which the critical spacing scales with about half of the viewing eccentricity independent of size, pass the crowding test^8,9,17^. However, recent studies have shown the importance of contextual modulations in crowding processing and that flankers outside Bouma’s window can increase or decrease crowding (cf. review of Herzog *et al*.^18^). For example, simple objects perception, such as a facial feature are crowding immune, while complex object identification tasks, e.g. the identification of a whole face, are crowding susceptible^9,10,17^. In summary, although there’s a consensus about the importance of the critical spacing and context as modulating factors in crowding processing, current models are still unable to surely predict which task and stimuli will crowd^19^. Thus, objects and tasks can only be defined crowding-susceptible if they pass the diagnostic criteria for crowding^8,9,17^.

When observers identify an object, the integration field that matches the object size is selected. If the smallest available integration field is too large to isolate the target, crowding occurs. Using a large tile, when a finer size is available, produces grouping^9,11,20,21^. Based on psychophysics, recent attempts have been made to isolate the involvement of different brain regions in crowding^22–28^. Freeman and Simoncelli^29^ modeled stimuli to match the integration fields characteristics and the receptive fields size increase with eccentricity and brain area. The Authors identified the observers’ crowding metamers, physically different but perceptually indistinguishable. Results indicate that metamers match the receptive field properties or area V2^29^.

Few neurophysiological studies have investigated neural correlates of the perceptual integration mechanism involved in crowding^22–28,30–32^. The current debate seems to focus on the early recruitment of visual areas through feed-forward processing^26,27^ as compared to through feed-back from higher-order visual areas^22,24,25^. On this matter, the role of attention in modulating the BOLD signal is crucial, and the response evoked during an unattended condition might provide some evidence of bottom-up processing^26^. However, except for Freeman *et al*.^24^, that used a highly demanding fixation task as unattended condition, attentional manipulations in other studies seem to be too weak to exclude that some resource may still be available for the peripheral stimuli^33–35^.

**Figure 1:**
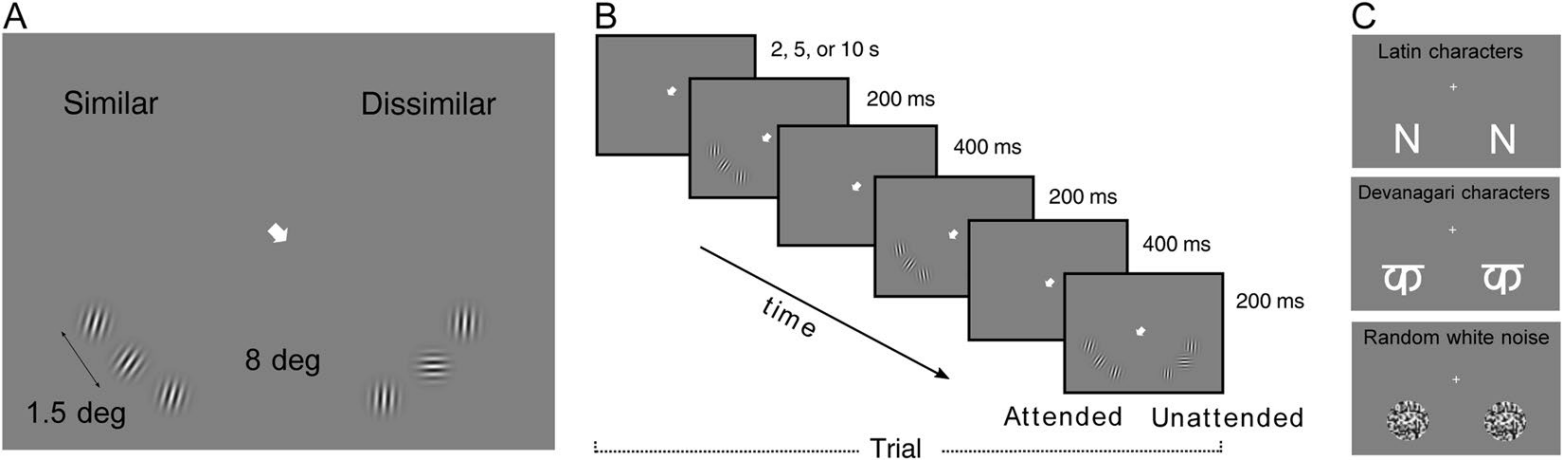
Design of the crowding and localizer experiments. (**A**) The panel shows an example of Gabor patches configurations (a central target with two flankers on each side): strongly crowded (left) and weakly crowded (right). In the strongly-crowded condition (left), the flanker patches have an orientation similar to the central patch (i.e., the orientation difference is less than 40 deg) while in the weakly-crowded condition (right) the flanker patches have an orientation remarkably different to the central patch (i.e., the orientation difference is more than 60 deg). For each quadrant, keep the figure at about 20 cm of distance, fixate the arrow and try to discriminate the orientation of the target in relation to the flankers. In the left quadrant, it is difficult to perform the discrimination task. The Gabor patches were simultaneously presented with equal eccentricity (8 deg) in a radial configuration. The distance between target and flankers was kept constant (1.5 deg center-to-center). (**B**) The panel shows the structure of a trial. Subjects were instructed to focus their attention on the three serially presented targets in the attended hemifield while ignoring the flanker distractors and the stimuli appearing in the other hemifield. Subjects were given a three-alternative, forced-choice task on the target (all the same, one different, all different). (**C**) Examples of stimuli in the three conditions of the localizer scan: Latin character (upper panel), Devanagari character (central panel) and dynamic white noise (lower panel). (Note that Gabor patches and localizer stimuli had the same envelope size and localizer stimuli were presented at the target location in both left and right lower quarter fields).

The goal of this study is to investigate whether different attentional conditions modulate crowding fMRI BOLD responses. To this end, we designed a three-alternative forced-choice 3-back task with a rapid presentation sequence. In relation to one single-trial (0-back task), this procedure has the advantage of increasing the stimulation while maintaining a rapid presentation to discourage eye movements. Three sequentially-presented triplets of Gabor patches (a central target with two flankers on each side) were presented simultaneously in the two lower hemifields at equivalent locations (Fig. 1A). The target orientation was selected randomly across trials. Visual crowding was manipulated by changing the difference in orientation between the three Gabor patches, and the distance between target and flankers was kept constant. Crowding condition (similar vs. dissimilar) was pseudorandomly varied across trials and independently in the two hemifields. Subjects performed a three-alternative forced-choice task about the target (all three targets are the same, one is different, all are different). Thus, in this paradigm the orientation of the flankers is not diagnostic for the task and subjects are required to isolate the target to succeed. In each trial, a central arrow indicating the side of the screen to attend was presented prior to the display sequence at the fixation location. The arrow remained on the screen throughout the sequence. Subjects were instructed to maintain fixation on the cross/arrow, to pay attention and respond to the location of the target to which the arrow was pointing, and to ignore the rest (the flankers and the stimuli in the unattended hemifield). Thus, the activity related to the “attended” condition, corresponding to the hemifield where the subjects were performing the task, was recorded together with the activity elicited by the “unattended” hemifield. Subjective reports indicate that observers were unaware of what was happening in the unattended hemifield (Fig. 1B).

Prior to the fMRI session, subjects underwent two sessions of psychophysical training outside the scanner in two separate days. Performance outside and inside the scanner confirmed that our manipulation was effective in deeply impairing performance under crowding conditions (similar orientation).

To study the BOLD response to these stimuli, we identified the borders between the early visual areas with standard retinotopic mapping methods and wide-field retinotopic stimulation that has been described in previous studies^36,37^. We also used a dedicated localizer scan to map the portion of visual cortex corresponding to the retinal position of the stimuli in the crowding experiment, and the visual word form area (VWFA) (Fig. 1B). Thus, the regions of interest were restricted to those voxels roughly corresponding to the location of the stimuli.

## Results

### Behavioral crowding effect

**Figure 2:**
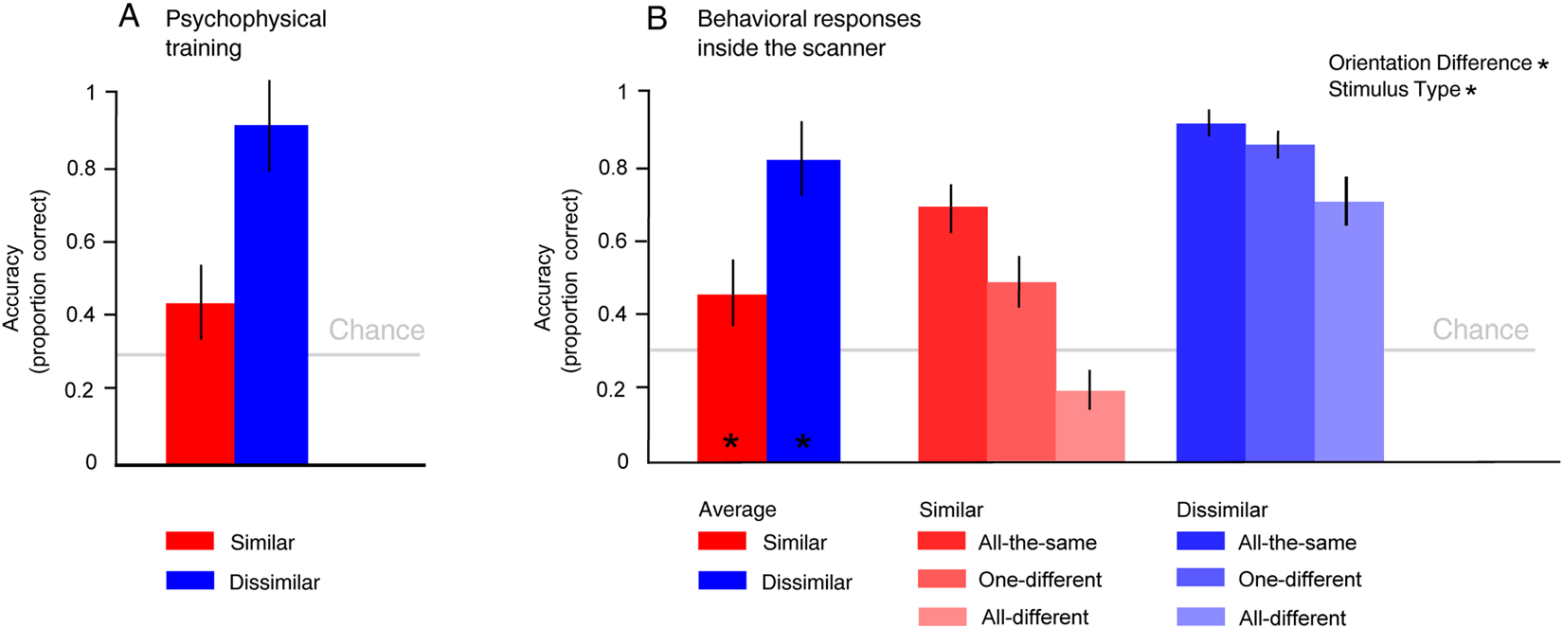
Behavioural effects of visual crowding. (**A**) Psychophysical training sessions. Graph bars show the identification accuracy, averaged across subjects (N = 12), for similar and dissimilar Gabor patches in the attended condition. (**B**) Behavioral responses inside the scanner. Data are plotted averaged (left) and separated according to the stimulus type – all the same, one different, all different (right). Chance level is at 33% (three-alternative forced choice). Error bars represent ± SD (*p_Bonferroni_ < 0.05).

Discrimination accuracy was plotted separately for behavioral data acquired during psychophysical training (Fig. 2A) and inside the MR scanner (Fig. 2B). Note that by design there was no measurable performance in the unattended condition. In both cases (psychophysical training and inside the MR scanner), in the attended condition, presenting Gabor patches with a similar orientation to the target induced a crowding effect that impaired the identification of the target. Similar results were obtained outside the scanner with letters in the spaced and unspaced conditions (M = 0.86, SD = 0.07 and M = 0.48, SD = 0.11 respectively).

For the twelve subjects who participated to the fMRI experiment, the accuracy inside the scanner for the dissimilar condition (M = 0.83, SD = 0.09) was higher than for the similar condition (M = 0.46, SD = 0.12) [*t*_*11*_ = 10.92, p = 3.038e-07, Cohen’s *d* = 3.45]. The performance of the subjects was higher than chance [similar vs chance: *t*_*11*_ = 3.75, p = 0.003; dissimilar vs chance *t*_*11*_ = 17.99, p = 1.66e-09], suggesting that participants could perform the task.

We tested the statistical significance of the results by performing a two-way repeated-measures ANOVA with Orientation Difference (similar/dissimilar) and Stimulus Type (all-the-same/one-different/all-different) as factors. The ANOVA showed a significant main effect of Orientation Difference [F_1,11_ = 173.62, p = 8.62e-06] and Stimulus Type [*F*_2,23_ = 15.24, *p* = 0.0001], but no significant interaction between the two factors. Paired *t*-test showed an effect of all-different stimulus type [all-different vs all-the-same: *t*_*11*_ = 0.78, p = 0.0001; all-different vs one-different *t*_*11*_ = 0.68, p = 0.004, Bonferroni corrected]. It should be noted that the lower accuracy for the all-different condition is a well-established phenomenon in the literature regarding the N-back task paradigm^38,39^.

#### Eye-movements recordings

The eye-movement data collected during the fMRI scanning indicate that subjects accurately maintained fixation while performing the crowding and localizer experiments. The fixation stability of a subset of subjects (6 out of 12), for which reliable eye data were recorded (see Methods), was excellent (always closer than 1 deg from the center of the fixation cross) and in line with standard parameters (e.g.^40–42^).

### Definition of retinotopic visual regions of interest (ROIs)

**Figure 3:**
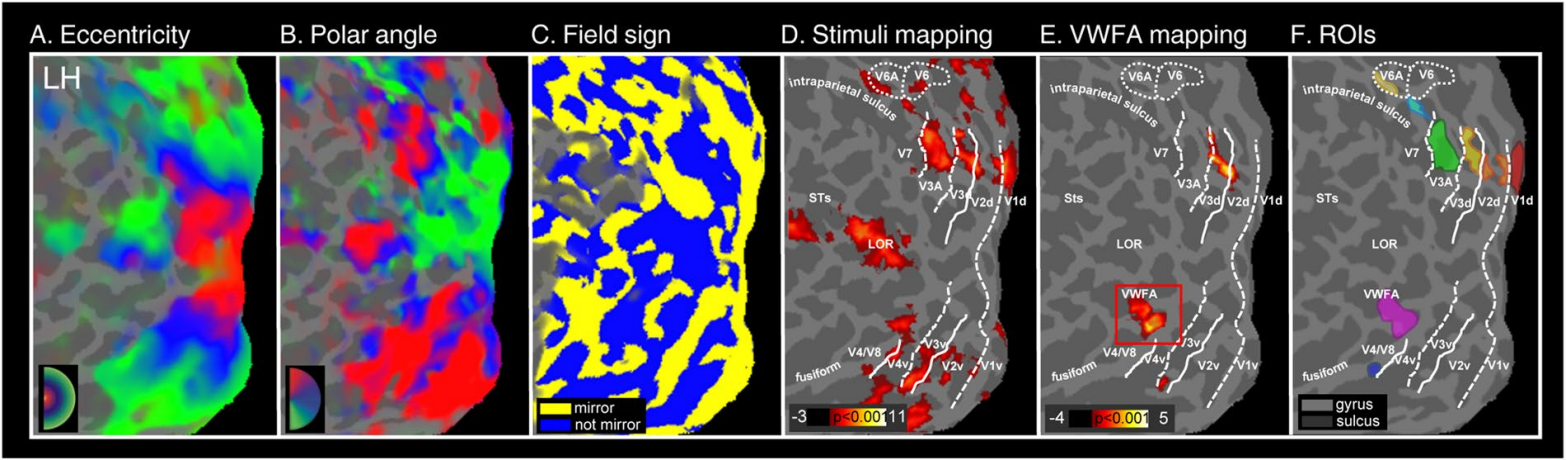
Retinotopic mapping and regions of interest. The figure shows a flattened representation of the posterior portion of the left hemisphere in a representative subject, overlaid with the results from the Retinotopic Experiment (**A**–**C**), the Localizer Experiment (**D**,**E**) and the resulting regions of interest (**F**). (**A**) Eccentricity map. Figure shows color plots of the response to a wide-field ring stimulus expanding at a constant slow speed (about 1°/s). Color hue indicates the response phase, which is proportional to the eccentricity of the local visual field representation. (**B**) Polar angle map. The color hue indicates the response phase proportional to the polar angle of the local visual field representation. Red, blue, and green areas represent preference for upper, middle, and lower parts of the contralateral visual field, respectively. (**C**) Map of retinotopic field sign. Analysis of retinotopic data (polar angle and eccentricity) by visual field sign (mirror-image versus non-mirror-image visual field representation). Mirror-image areas (yellow; e.g., V1), and non-mirror-image areas (blue; e.g., V2) are shown. (**D**) Target-specific regions were localized in each subject by presenting dynamic white noise, during the localizer experiment, in the same position and size of the target (p_FDR_ < 0.001). The white lines on the surface show the borders between retinotopic visual areas. Specifically, the dotted and solid lines indicate vertical and horizontal meridians, respectively; the enclosed dotted lines indicate V6 and V6Av borders. (**E**) The visual word form area (VWFA, in the red box), defined in each subject, in the left fusiform gyrus, by the contrast between Latin Vs Devenagari characters (p_FDR_ < 0.001). (**F**) The regions of interest, represented in different colors, were restricted to those voxels in the early visual areas that respond to the lower quadrants of the visual field: V1d, V2d, V3, V3A, V7, V6Av, V4/V8, and all the voxels in the VWFA defined by the localizer. Major sulci and gyri (dark gray and light gray respectively) are labeled as follows: intraparietal sulcus, STs (Superior Temporal sulcus); LOR (Lateral Occipital Region); fusiform (fusiform gyrus).

Figure 3 shows the results of the retinotopic mapping experiment and the associated ROI definition in the hemisphere of a representative participant. As described in the Methods, wide-field retinotopic mapping allowed to define the borders between striate and extrastriate visual areas (see Fig. 3A–C). The eight lower-field visual region (V1d, V2d, V3, V3A, V7, V6, V6Av, and V4/V8) were successfully defined in all 24 hemispheres. We then mapped the portion of each of these visual regions that was activated by the dynamic white-noise localizer (Fig. 3D). BOLD activity was not present in all visual areas of all analyzed hemispheres. Thus, the number of resulting ROIs on which we performed the main analysis (see below) was variable across regions. We identified activation in V1d in all 24 hemispheres, in V2d and V3A in 23 hemispheres, in V3 in 22 hemispheres, in V4/V8 in 20 hemispheres, in V7 in 17 hemispheres, in V6Av in 8 hemispheres, and in V6 in 5 hemispheres. Given the low number of V6 maps that we could map in each subject, we excluded area V6 from any further analysis. The VWFA was instead defined using the letter conditions in the localizer scans (Fig. 3E) and was successfully identified in 10/12 left hemispheres. Next, we intersected the map derived from the localizer with the lower representation of the retinotopic regions to define a set of ROIs. Each ROI included the set of surface vertices within each retinotopic region that was significantly activated by the dynamic white-noise stimulus (Fig. 3F).

### Increased activity during strongly-crowded condition

**Figure 4:**
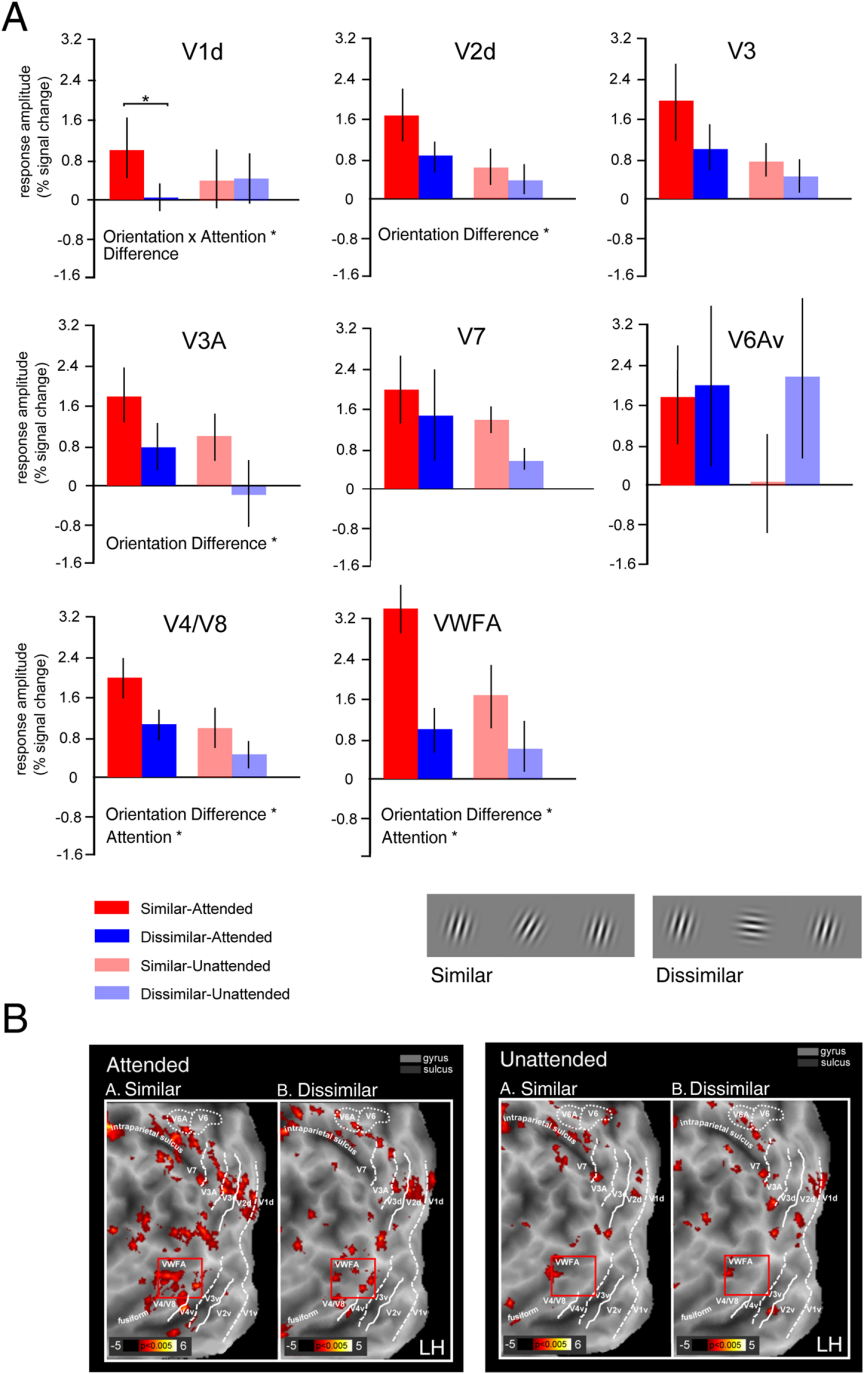
Average BOLD response amplitude for Similar-Attended, Dissimilar-Attended, Similar-Unattended, and Dissimilar-Unattended. (**A**) Graph bars indicate BOLD signal change in the similar and dissimilar conditions separately for attended and unattended conditions, in cortical areas V1d, V2d, V3, V3A, V7, V6Av, V4/V8, and VWFA. Error bars represent ± SEM (*p_Bonferroni_ < 0.05). (**B**) The figure shows four flattened representations of the posterior part of the left hemisphere of one representative subject, overlaid with activation maps from the Gabor (similar and dissimilar) in the attended and unattended conditions. The white lines on the surface show the borders between the retinotopic visual areas. The dotted and solid lines indicate vertical and horizontal meridians, respectively. The red square indicates the position of the VWFA as defined by functional localizer (see methods for details). The inset shows a schematic representation of the Gabor stimuli we used in the main fMRI experiment to produce similar and dissimilar conditions.

We induced crowding by manipulating differences in orientation between the target and the flanker patches: lower differences in orientation produced a performance impairment in orientation discrimination. The stimulus array evoked a robust BOLD activity in all the ROIs (V1d, V2d, V3, V3A, V7, V6Av, V4/V8, and VWFA). We assessed the crowding and the attentional effects, by performing a repeated-measures ANOVA with Attention (attended vs. unattended) and Orientation Difference (similar vs. dissimilar) as factors. The results are shown in Fig. 4A.

The ANOVA yielded a significant two-way interaction between Orientation Difference and Attention in V1d [*F*_1,23_ = 10.984, *p* = 0.006]. Post-hoc comparisons revealed that only in the attended condition the BOLD signal in V1d was greater in the similar than in the dissimilar condition (*p = *0.006).

Two visual areas showed a main effect of Attention: V4/V8 [*F*_1,19_ = 12,001, *p* = 0.002] and VWFA [*F*_*1,9*_ = 7,480, *p* = 0.023]. In both areas, the mean signal change was stronger in the attended than in the unattended condition. Several visual areas showed a main effect of Orientation Difference: V2d [*F*_*1,22*_ = 5,389, *p* = 0.029], V3A [*F*_*1,22*_ = 10,086, *p* = 0.004], V4/V8 [*F*_1,19_ = 15,526, *p* = 0.0008], and VWFA [*F*_*1,9*_ = 12,826, *p* = 0.005]. All these areas exhibited a stronger BOLD response in the similar than in the dissimilar condition.

Figure 4B shows the fMRI results in the left hemisphere of one representative participant. Activation in the four conditions, resulting from the combination of Attention and Orientation Difference, is displayed together with a set of lines representing the boundaries of early visual areas. Figure 4B also shows the position in this subject of the functionally identified VWFA area, located in the fusiform gyrus, posteriorly to V4/V8. While the two maps in the unattended condition are quite overlapping, there are evident differences in the attended condition. In this condition, there is an overall increase in the fMRI activation for the similar Gabor patches.

### Control analysis: Crowding effect did not depend on the ROIs size

**Figure 5:**
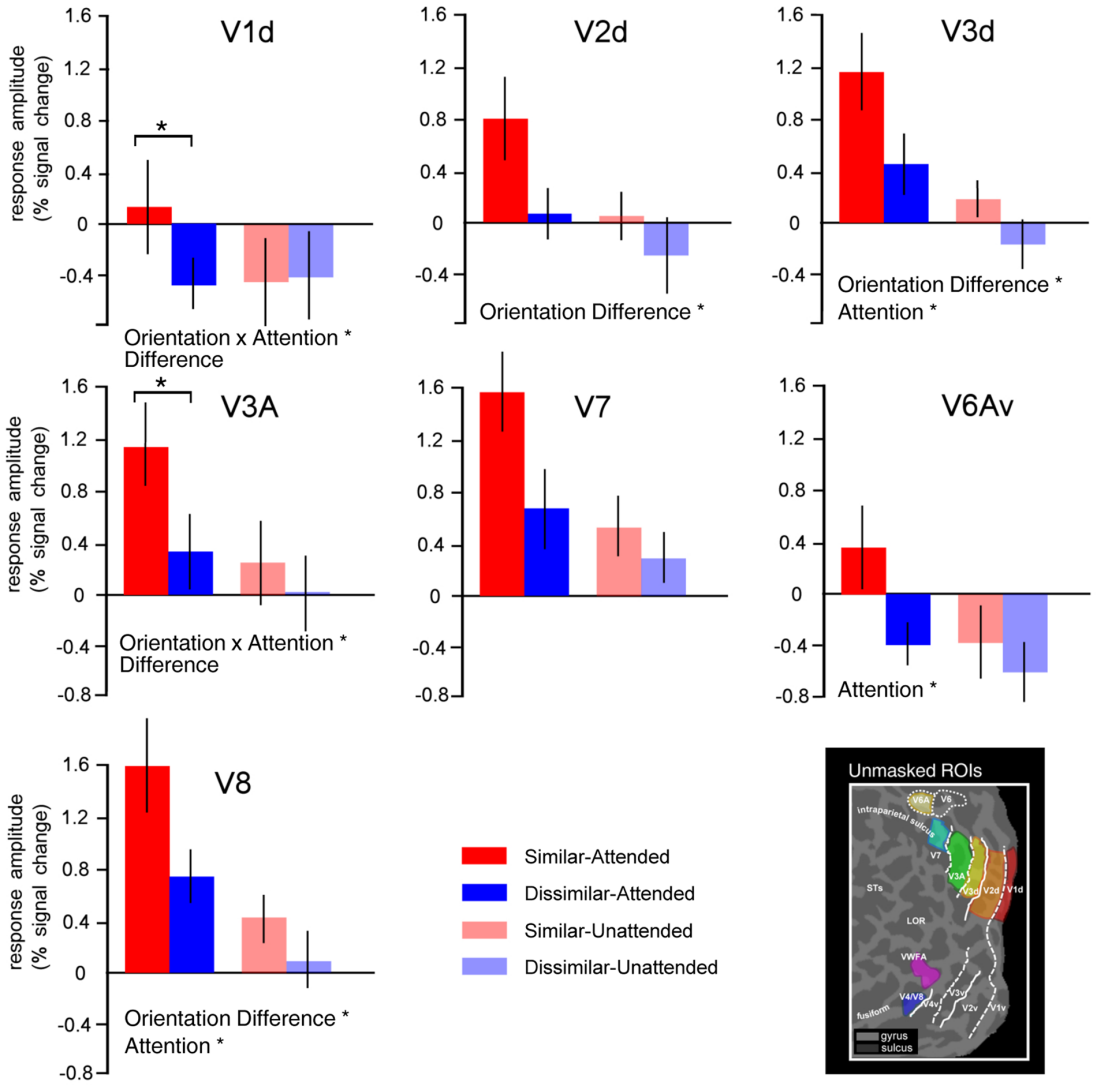
Average BOLD response amplitude for Similar-Attended, Dissimilar-Attended, Similar-Unattended, and Dissimilar-Unattended in the unmasked ROIs. Graph bars indicate BOLD signal change in the similar and dissimilar conditions separately for attended and unattended conditions, in unmasked cortical areas V1d, V2d, V3, V3A, V7, V6Av, and V4/V8. Error bars represent ± SEM (*p_Bonferroni_ < 0.05). On the bottom, a right panel shows a flattened representation of the posterior part of the left hemisphere of one representative subject, overlaid with the unmasked retinotopic visual areas used in this analysis.

We measured the crowding effect in ROIs created by masking the localizer map with the dorsal representations of the retinotopic visual regions representing the lower quadrants. These regions were restricted to the target location. However, a potential concern is that some part of the signal related to the flankers was included in the sampled ROIs and that the crowding effect we observed was related to the ROI size. In fact, due to the small amount of applied spatial smoothing and the spatial point spread function intrinsic to the magnet, it is possible that some voxels related to the flankers and to  non-stimulated cortex might have been introduced in the analysis (see Methods). Therefore, we re-ran the analysis on the unmasked retinotopic regions representing the lower visual quadrants to check for this possible confound (Fig. 5) Note that the analysis was not repeated in the VWFA since for this region we used an independent localizer.

Overall, the pattern of results was similar to the one obtained using the masked regions, with an increased activity in the similar attended condition. As expected, BOLD activity was greatly reduced (by about a half) in relation to the activity extracted in the masked regions, probably due to some lateral inhibition and intrinsic spontaneous activity which may be greatest source of correlated noise^43^.

We assessed the crowding and the attentional effects, by performing a repeated-measures ANOVA with factors Attention (attended vs. unattended) and Orientation Difference (similar vs. dissimilar). Only two areas showed a significant two-way interaction between Attention and Orientation Difference: V1d [*F*_*1,23*_ = 10.984, *p* = 0.006] and V3A [*F*_*1,23*_ = 7.46, *p* = 0.019]. Post-hoc comparisons revealed in these two regions, in the attended condition, that the BOLD signal was greater in the similar than in the dissimilar condition (V1d, p = 0.006; V3A p = 0.001).

Three visual areas showed a main effect of Attention: V3d [*F*_*1,23*_ = 5.54, *p* = 0.038], V6A [*F*_*1,9*_ = 5.80, *p* = 0.04], and V4/V8 [*F*_*1,23*_ = 14,10, *p* = 0.003]. In all three areas, the mean signal change was stronger in the attended than in the unattended condition. Several visual areas showed a main effect of Orientation Difference: V2d [*F*_*1,22*_ = 8.48, *p* = 0.01], V3d [*F*_*1,22*_ = 13.75, *p* = 0.003], and V4/V8 [*F*_*1,19s*_ = 17.12, *p* = 0.001]. All these areas exhibited a stronger BOLD response in the similar than in the dissimilar condition.

## Discussion

Beyond acuity, crowding imposes a substantial limit on the ability to identify peripheral objects or tiny letters seen foveally. It is a well-defined psychophysical phenomenon that fundamentally constrains visual recognition models.

Here we used a combination of event-related fMRI measurements, functional localizers, and wide-field retinotopic mapping to investigate the effect of attentional modulation on the BOLD response in human visual cortex under crowding conditions. Visual crowding was manipulated by changing the orientation of the Gabor patches. Inside and outside the scanner, the behavioral results showed a strong crowding effect. To study BOLD activity associated with these stimuli, we mapped seven retinotopic visual areas (V1d, V2d, V3, V3A, V7, V4/V8, and V6Av) through wide-field retinotopic mapping^36,37^, and the visual word form area (VWFA) by a dedicated functional localizer for familiar letters, comparing Latin and Devanagari characters^24,44^. We chose a paradigm similar to the one proposed by Baker *et al*.^44^ because more coherent with our main experiment. Other studies identified the VWFA based on responses to visual words^45^. Based on another ad-hoc visual localizer, we then defined a region within each visual area retinotopically corresponding to the stimulus position^23,25–27^. We manipulated awareness of stimuli with an overt attentional cueing paradigm to evaluate whether early activity related to crowding might be due to an attentional modulation acting through feedback connections from higher-level areas. Within this cueing paradigm, attention is inhibited in the contralateral hemifield^33–35^. Our subjects reported being completely unaware of what was presented in the unattended hemifield. Thus, we believe that our manipulation brings us as close as possible to the evaluation of perception without attention.

### The crowding network

Gabor patches are stimuli particularly suited to characterize the low-level properties of vision^46–48^. They have a tight spectrum, characterized by just one feature and one orientation. They are used in visual neuroscience since they efficiently activate neurons in the visual cortex. Coherently, we found that this type of stimulus was very effective in driving the visual cortex, evoking a general robust BOLD activity in most of the tested visual areas that retinotopically represent the locations of the stimuli.

By examining response amplitude associated with different crowding conditions and attentional modulation, we found that the perceptual processes underlying the crowding phenomenon were not localized in a single region and extended across a wide cortical network (Figs 4 and 5). In fact, we observed that crowding was effective in modulating the activity of areas V1d, V2d, V3A, V4/V8, and VWFA. The crowding phenomenon thus involved the striate primary visual area as well as the extrastriate cortex. Our results indicate that the crowding relevant regions are not confined to the ventral visual stream tested in other studies but include almost all the dorsal visual areas (except for the higher-level extrastriate areas V7 and V6A, see Fig. 4). Albeit we used Gabor patches, we found that also the ventral VWFA, located in the left lateral occipito-temporal sulcus and responsive to words and reading^45,49,50^, was differentially modulated by crowded and non-crowded conditions.

Attention modulated fMRI BOLD responses in visual areas V1d, V3A, V4/V8 and VWFA (Fig. 4). While areas V3A, V4/V8 and VWFA, showed a general main effect of attention (i.e., unrelated to the crowding condition), V1d exhibited a different behavior. In fact, we found that crowding modulated the activity in V1d only when subjects were attending to the target.

In our paradigm, while the subjects were fixating the central cue, the attention was overtly directed to the peripheral location. Thus, the unattended condition corresponded to the activity related to the stimuli presented simultaneously in the opposite hemifield. The unattended stimuli received an attentional inhibition from the contralateral location^33–35^. With this paradigm we found that crowding modulates the early visual areas only when subjects were required to attend to the stimuli and to perform the task. Similarly, Chen *et al*.^27^ showed that when subjects were actively processing the target, the BOLD response was significantly suppressed in all the early visual areas. However, when there was no attention to the stimuli they found no suppression. This result may suggest that crowding processing could be triggered through feedback connections from higher-order areas, rather than being automatic and bottom-up.

### The effect of crowding: suppression or enhancement?

In the last decade, many psychophysical studies have converged on the finding that simple models of crowding cannot fully explain the phenomenon^18,51–53^. To explain the complex interaction between target and flankers, these studies have suggested that crowding and grouping rely on the same integration mechanism^21,52,54^.

Ultimately, grouping and crowding are intrinsically related to two factors: the type of task and the nature of the perceptual experience. In crowding experiments subjects’ task typically involves the identification of the target or the discrimination of one of its properties. Consequently, the target needs to be “isolated” from the flankers. Alternatively, the task can involve some computation to be performed on both target and flankers, so they must be “integrated” or bound together (e.g. Parkes *et al*.^55^). Thus, features integration over a large area including both target and flankers may help in grouping and impair performance in crowding. The perceptual experience is also influenced by the stimuli and can be characterized by a coherent and stable percept (as in grouping of similarly aligned elements), or by an unstable jumble of features (as in flanked letter identification).

Based on the type of task and perceptual experience, flankers may play opposite roles^54^. For example, bound flankers help grouping but may induce crowding. Spatial relations between flankers and between target and flankers modulate the perceptual experience, and thus the level of grouping/crowding^18,51^. In psychophysical experiments such as the one reported by Parkes *et al*.^55^ Gabors may be organized as to fall within the same integration region (i.e., similarly oriented, closely spaced and peripherally presented as in our crowded condition). Under these conditions, target isolation fails and target and flankers are combined or grouped. If the subject is required to isolate the target to identify its orientation, the task is experienced as hard, and the subject fails (i.e., large target orientation thresholds)^55^. We may call this phenomenon “crowding.” If the subject is required to tell the overall pattern orientation, the subject succeeds and the task becomes easier as a function of the number of similarly oriented Gabors (i.e. small orientation thresholds)^55^. We may call this phenomenon “grouping.” Both phenomena are explained by the same integration mechanism, namely, according to Parkes *et al*., by compulsory averaging within the integration region with no signal loss^55^.

In our experiment, we asked subjects to pay attention to the target and to perform an orientation discrimination on the target only. In other words, to perform the task subjects needed to “isolate” the target from the flankers. However, because of the intrinsic properties of the stimuli, in the crowding condition target and flankers perceptually “grouped”, thus impairing the performance. In this condition, we found an increased activity compared to the uncrowded condition. In particular, we found an early enhancement mediated by attention and a later response independent of attention. An increase in cortical activation with Gabor patches in similar conditions was already reported in one study^56^ in the framework of the biased competition theory. According to this theory, competitive interactions between visual stimuli are modulated by attention and bottom-up factors like orientation similarity. In this context, within a perceptual group, (for example Gabor patches with identical or similar orientations), the stimulus similarity releases from competition interactions. In this case, the theory predicts an enhancement of the neural activity. Conversely, between perceptual groups, (for example Gabor patches with different orientations), the suppressive interaction between the stimuli cause a reduction of the neural activity^56–58^.

Our results did not depend on the size of the sampled ROIs. We run a control analysis on the unmasked retinotopic regions, in which stimulated and not stimulated portions of the cortex were included in the analysis. We found the same trend, an increased activity during the strongly-crowded condition, in all visual areas even though with a much lower evoked BOLD response.

Our crowding effect cannot be explained by eye-movement artifacts. The subjects were trained psychophysical observers, and the off-line analysis of the eye-movements confirms that subjects maintained a stable fixation during the visual stimulation. Thus, the effect we observed is unlikely to be the result of experimental confounds resulting from the stimuli.

However, our results are different from those found in the majority of previous neuroimaging studies, in which the crowding conditions were typically associated with a reduced activity or no difference compared to the uncrowded conditions in the early visual areas. There is some evidence that the type of task plays a crucial role in crowding processing and might explain the discrepancy across studies. Anderson *et al*.^25^ found in the crowding condition a reduced BOLD activity in all visual areas beyond V1 with an adaptation task but an enhancement tendency when the adaptation paradigm was removed. In addition, Freeman *et al*.^24^ using a fixation task showed that differences in the mean amplitude across crowded and uncrowded conditions did not correlate with crowding. The authors found a significant reduction in the BOLD activity in a crowded condition with an array of letters as well as in an uncrowded condition with an array of Gabor patches.

Overall, the variability across studies cautions against a univocal interpretation of the direction of the effect. Nonetheless, it suggests that other factors, such as the attention, the type of task, the phenomenological percept, and the internal state of the subject may play an important role, and they differently affect the BOLD response, similarly to what has been shown in psychophysical studies^12^.

In crowding experiments, the relation between BOLD response, cognitive task and performance is complicated by the interplay between crowding and grouping, two intrinsically-related phenomena probably belonging to the same integration mechanism^55^. The relationship between these two phenomena and the resulting phenomenological experience make it difficult to predict the direction of the effect a priori. Indeed, some recent studies have shown conflictual results: cortical response in early visual areas to grouped coherent contours relative to scrambled elements has been reported as both an enhancement^59–61^ as well as suppression^62,63^. More extensive studies are needed to isolate the contribution of the cognitive task, stimuli, perceptual experience, performance, and the internal state of the subject on the BOLD activity associated with the crowding and grouping phenomena.

### Early or late locus of visual crowding?

There is a general agreement on locating the crowding correlate somewhere in the visual cortex beyond the site of the binocular combination, based on the observation that visual crowding occurs even when target and flankers are shown in dichoptic presentation^6^. However, whether this phenomenon occurs as early as V1 is still debated. Some psychophysical and neurophysiological studies suggest that the earliest visual area that is affected by crowding may be V1^25–28^. The progressive increase in crowding modulation that has been found from early to higher visual areas seems to correlate with the increase of the receptive field size from V1 to V4^20^. Thus, the effect of crowding in V1 might depend on the target-flankers pooling occurring in higher visual areas that modulate V1 through feedback connections. Jehee and colleagues^64^ proposed a model in which crowding effect does not occur at a specific level in the neural system, but it is the result of a re-entrant circuit, which starts in V1 and projects to some higher visual areas. According to this model, the recognition of an object involves recurrent interactions across multiple levels of the cortical visual hierarchy^24^. Our results are compatible with this hypothesis. In fact, we found that in V1 crowding modulation was effective only in the attended condition. The first areas that start to show some hint of bottom-up modulation are V2/V3, consistently with a study that found in V2 the critical receptive fields size to induce visual crowding^29^.

It has been speculated that V4/V8 could also be the locus of visual crowding for different compelling reasons. In this area, the receptive fields are big enough to include target and flankers^65^. Furthermore, the anisotropy of about 2:1 and the receptive field size^66^ match the radial/tangential anisotropy of crowding^67^. Moreover, V4/V8 combines information from different stimulus types^68,69^, in agreement with the finding that visual crowding is modulated by different stimulus categories^9,17^. Consistently with this hypothesis, we observed an increased activity during crowding condition in V3A, V4/V8, and VWFA, where supposedly receptive fields are large enough to include target and flankers. In fact, at 8 degrees of eccentricity, receptive fields are about 6.5 deg in V3A and about 6 deg in V4/V8^68^, while the stimulus array (in the crowding conditions) covers an area of 1.5 × 3 deg. These results suggest that higher ventral areas might play an important role in crowding processing. Consistently, Freeman *et al*.^24^ found that crowding was associated with a reduction in the temporal correlations between early visual areas and VWFA. Importantly, using stimuli other than letters we found that the VWFA was modulated by crowding. This result has important implications for characterizing the VWFA and its role in perceptual integration. The choice to use Gabor patches instead of letters has an important advantage. Indeed, using Gabor patches rules out the possibility that the observed modulation is stimulus-specific and suggests that this region might have a cross-category function. Consistently, recent studies have shown that the VWFA over and above being a stimulus-specific region has more general processing properties related to the stimulus complexity and the objects’ “group-ability”^70,71^.

Complementary support to the critical role of the occipito-temporal cortex on crowding processing comes from neuropsychological studies. Strappini *et al*.^72^ found that visual apperceptive agnosia, a deficit in recognizing familiar objects, may be explained by visual crowding. This finding has significant implication for constraining the neural correlates of crowding. Besides the variety in the size of lesion and severity of the perceptual deficit across patients, apperceptive agnosia is usually associated with either bilateral or unilateral right occipito-temporal lesions sparing striate cortex (V1) and the parietal areas^73^. It is possible to speculate that visual crowding in these patients might reflect limited plasticity in the occipito-temporal cortex to recover from the neural loss, i.e. insufficient recruitment of other neurons to entirely make up for the loss^72^.

Future studies, with appropriate paradigms, will be required for investigating whether visual crowding in normal subjects is specifically related to some region or to a broad cortical network involving the dorsal stream or the ventral stream which is usually impaired in these patients.

## Conclusions

Crowding is a well-defined psychophysical phenomenon that limits object recognition across the visual field. Behaviorally, it results in impaired recognition when the task is target identification, while enhanced performance is observed when observers are required to judge a texture^11,21^. This indicates that crowding reveals the core features of vision. Here we show that crowding involves a wide network, modulating the response of area V1d, V2d, V3A, V4/V8, and VWFA. Early V1 activity with strongly-crowded Gabor patches is registered only when attention is fully devoted to the target location. This indicates that V1 response to crowding might not be automatic nor due to bottom-up processing and provides support to the view of crowding as a mid-level visual phenomenon.

## Methods

### Participants

Twenty healthy adults participated in the study. Four subjects were discarded during the training session or the experiment because either had difficulties in keeping the fixation or in performing the task. Four additional subjects were discarded because they abandoned the study at different stages.

Each of the twelve participants underwent three separate functional magnetic resonance imaging (fMRI) acquisition sessions in separate days. The first session included the main crowding experiment, the second one the localizer session, and the last one the phase-encoded retinotopic mapping and the anatomical scans for the reconstruction of the cortical surface. All participants had normal or corrected-to-normal visual acuity (mean age 27 years, range 26–31, 1 female) and no history of a psychiatric, neurological or attentional disease. All participants had extensive experience in psychophysical and fMRI experiments and were paid for their participation. All participants gave written informed consent. All procedures were approved by the Ethics Committee of Fondazione Santa Lucia, Rome, Italy. All experiments were performed in accordance with relevant guidelines and regulations.

### Crowding experiment

In the main event-related fMRI experiment, hereafter crowding experiment, we investigated the phenomenon of visual crowding using Gabor patches, while manipulating the subjects’ attention so that stimuli inducing the crowding effect could appear at or away from the attentional focus (Fig. 1A,B).

In the psychophysical training the stimulus set in each hemifield could be composed either of Gabor patches, arranged so as either to produce or not to produce a crowding effect, or of arrays of letters, spaced and unspaced. Psychophysical comparison between letters (generally used in crowding) and Gabors ensures that the magnitude of the effect is comparable with what expected. To avoid the confounding role caused by the increased stimulated area due to spacing, only Gabor patches were included in the fMRI study.

Gabor patches were sinusoidal gratings with a spatial frequency of 3 cycles/degree windowed by a two-dimensional Gaussian, and subtending approximately 1.5 deg. Each pattern was composed of three Gabor patches (a central target with two flankers on each side) simultaneously presented in a circumferential configuration (Fig. 1A). Crowding was manipulated by changing the orientation of the flanker patches relative to the central patch (Fig. 1A). In the strongly-crowded condition (similar), the flanker patches had a similar orientation to the target (between 20 and 40 deg of rotation relative to the target orientation), resulting in poor discrimination of the target orientation at the given eccentricity. In the weakly-crowded condition (dissimilar), the flanker patches had a different orientation to the target (between 60 and 90 degrees of rotation relative to the target orientation), resulting in high accuracy in discriminating the target orientation. The orientation of target and flankers could vary in each display while both flankers always had the same orientation. The orientations were randomized across trials. The spacing between target and flanker patches was 1.5 degrees center-to-center. All stimuli were drawn in white on a gray background (uniform gray 128 with a luminance of 45 cd/m^2^) in the lower visual field at an equal eccentricity of 8 deg of visual angle. We located visual stimuli in the lower screen because there is some evidence that crowding in the lower visual field is stronger than in the upper visual field^74^.

In each trial, attention was directed either to the left or to the right hemifield, while two sets of stimuli were simultaneously presented, one in the attended and one in the unattended hemifield, and subjects performed a discrimination task on the stimuli in the attended hemifield. A white arrow pointing at either the lower left or the lower right visual field was always visible at the center of the screen and served both as a fixation point and as a cue to direct the participant’s attention to the corresponding hemifield. A trial consisted of the simultaneous presentation in the two hemifields of a sequence of three consecutive stimulus patterns, for 200 ms each, spaced 400 ms apart (Fig. 1B). Participants were instructed to focus their attention only on the *central* target position of the attended hemifield while ignoring the flanker distractors and the stimuli appearing in the other hemifield. Participant performed a three-alternative forced-choice task, deciding whether the three serially presented central elements were all the same, or if they were all different, or two of them were the same and the third one different and pressed the corresponding response button with the right hand. We chose to manipulate crowding by changing the orientation difference of the stimuli rather than the presentation timing. This manipulation was meant to avoid possible confounds caused by the sequential presentation which presumably induces apparent motion.

The experimental design was 2-by-2 factorial, with factors Attention (attended, unattended) and Orientation Difference (similar, dissimilar).

The main experiment consisted of 8 acquisition runs, each composed of nine blocks of trials, separated by a variable fixation period of either 2, 5, or 10 s. The Attention factor (i.e., the side pointed at by the central arrow) was alternated across consecutive blocks, while the Orientation Difference condition (similar vs. dissimilar) was pseudorandomly varied across trials and independently in the two hemifields. Each block included six trials spaced 4.8 s apart. Overall, each participant performed 216 experimental trials.

To familiarize with the task and to reach a certain performance criterion, we performed a preliminary behavioral training session. Each subject was behaviorally trained outside the scanner in two separate sessions one week and two weeks before the fMRI main experiment session. Visual stimuli were presented on a 15” computer display that subtended the same degrees of visual angle as in the fMRI scanner. Subjects were seated in front of the display with the head mechanically stabilized with a chin rest. Subjects were asked to perform the same crowding experiment, subdivided, as the main experiment, in 8 different blocks. Subjects were allowed to rest between consecutive blocks. We verified that during the training session the participants’ performance was stable and that they reached the learning criterion of 80% accuracy for the dissimilar condition. Four subjects were discarded during the training session because either had difficulties in keeping the fixation or in performing the task. Before scanning, subjects were again briefly trained inside the scanner with a preliminary warm-up session.

### Visual localizer

We used a dedicated localizer scan to map at the same time the portion of visual cortex corresponding to the retinal position of the stimuli in the crowding experiment, and the visual word form area (VWFA)^49^_._ We included the VWFA in this study because recent neuropsychological evidence indicates that the occipito-temporal cortex may be a critical locus for visual crowding^72,75^. Moreover, there is a strong indication that VWFA is modulated by crowding conditions (e.g.^24^). However, in these fMRI studies, VWFA has been tested only with letters. Here, we tested the hypothesis that this area has a more general cross-category function, i.e. that it is modulated by crowding regardless the type of stimulus.

During this experiment, we presented three types of stimuli at the same retinal positions used for the targets of the crowding experiment: Latin characters, Devanagari characters, and dynamic white noise (Fig. 1C). Stimuli were drawn in white on a gray background. Subjects were instructed to look at the fixation cross centered in the middle of the screen while eye movements were recorded during the session.

Latin characters were upper-case letters in the font Courier New. Devanagari Characters were lower-case characters in the Xdvng font. Both Latin and Devanagari characters subtended approximately 1.5° and matched for the overall spatial envelope. The noise consisted of independently generated square checks with luminance drawn randomly from a uniform distribution, which allows a greater range of crms (root mean square contrast) values than a Gaussian distribution^76^. The crms values of the noise ranged from 0.02 to 0.18 in steps of approximately 0.25 log units. Every check subtended about 0.2° and the duration of each noise check was one video frame at 60 Hz. The noise was presented into a circular window with 1.5° of diameter.

Stimuli were presented for 300 ms each, spaced 800 ms apart, and were arranged in 16 s blocks with twenty stimuli per block. Participants underwent four acquisition runs, each composed of sixteen blocks, including four blocks per condition and four fixation blocks. We mapped the VWFA area by contrasting Latin versus Hindi characters, and the position of the target stimulus (as used in the crowding experiment) by contrasting random dynamic-white-noise versus the fixation baseline.

### Wide field retinotopic mapping

We mapped responses to the polar angle (measured from the contralateral horizontal meridian around the center of gaze) and eccentricity (distance from the center-of-gaze) using standard phase-encoded retinotopic stimuli^36^. The stimuli were presented using a wide-field display^37,77,78^ and consisted of high contrast light/dark colored checks flickering in counter-phase at 8 Hz in either a wedge or a ring configuration (polar angle and eccentricity mapping, respectively) extending over 80 degrees of visual angle (see Experimental set-up for details). The average luminance of the stimuli was 105 cd/m^2^. The duration of one complete polar angle or eccentricity cycle was 64 s; 8 cycles were presented during each fMRI run. Each participant underwent two polar angle and two eccentricity runs. During wide-field retinotopic mapping, subjects were required only to maintain fixation on a central cross. The retinotopic mapping (polar angle and eccentricity) allowed us to define the boundaries of retinotopic visual areas on the cortical surface for each subject on the basis of the visual field sign (see Data Analyses for details^36^). The use of a wide-field retinotopic stimulation allowed us to define not only the traditional visual areas (V1d, V1v, V2d, V2v, V3, V3A, V7, VP, V4v and V4/V8) but also two dorsal visual areas, V6 and V6Av, recently mapped by our group^37,42^. While V6 responds to the entire contralateral hemifield^39^, area V6Av is a lower-only retinotopic region^42^. Since stimuli were presented in the lower visual field, we included in the analysis only the lower representations of these visual areas.

### Apparatus

The MR examinations were conducted at the Santa Lucia Foundation (Rome, Italy) on a 3T Siemens Allegra MR system (Siemens Medical Systems, Erlangen, Germany), with a standard transmit-receive birdcage head coil equipped for echo-planar imaging.

Stimuli were generated by control computers (a standard PC and an SGI O2, both equipped with a standard 3D graphics card) located outside the MR room. For the crowding experiment and the localizer scans, stimuli were presented with an in-house software, implemented in MATLAB (The MathWorks Inc., Natick, MA, USA) using Cogent 2000 (developed at FIL and ICN, UCL, London, UK) and Cogent Graphics (developed by John Romaya at the LON, Wellcome Department of Imaging Neuroscience, UCL, London, UK). For the retinotopic mapping, stimuli were generated using an in-house ×11/OpenGL program (original GL code by A. Dale, supported and extended by M. Sereno; Mapper software: http://kamares.ucsd.edu/~sereno/stim/ and a Tiga-diamond (Salient AT3000) graphics card.

An LCD video projector (Sharp GX-3800, 640 * 480 pixels, 60 Hz refresh) with a customized lens projected stimuli onto a back-projection screen attached to the back of the head coil. Head position was stabilized with foam padding.

For the retinotopic mapping experiment, we used a wide-field setup similar to that previously described^37^. To get a wide-field stimulation, we lowered the subject’s body by about 4 cm from iso-center so that the bottom portion of the screen was not blocked and we used an enlarged mirror so that the screen periphery was visible. The eye-to-screen light path was about 18 cm. At this short viewing distance, visual stimuli subtended up to 80 (±40) deg horizontally and 60 (±30) deg vertically. For the localizer and the crowding experiment, we used a standard setup where the average viewing distance was 66.5 cm, subtending a visual screen of 23 × 12 deg.

In the crowding experiment, in which the subject’s response was required, manual responses were collected using a magnet-compatible response pad connected to the control computer via optic fibers. Retinotopic and the localizer experiments used passive viewing and continuous central fixation throughout the period of scan acquisition. To measure fixation performance during the crowding and localizer experiments, the eye movements of the subjects were monitored using an MR compatible ASL eye tracking system (Applied Science Laboratories, Bedford, MA; Model 504, sampling rate 60 Hz). This system is fully MR-compatible and does not produce any artifact in the BOLD images. Fixation accuracy was quantified by calculating (in degrees of visual angle) the actual eye position with respect to the fixation cross. In addition, gaze position was also visually inspected online by an experimenter using the ASL eye-tracking screen to detect unwanted saccades during crowding and localizer experiments. The on-line control was used mainly to give subjects feedbacks on excessive blinking or sign of lapses in attention during the runs. When this happened, subjects were asked to repeat the run. Usable data was not obtained with every subject for the usual reasons - e.g., difficulty maintaining pupil tracking across the experiment with light-eyed subjects or with subjects wearing contact lenses. We obtained usable data only in six out of twelve subjects for which statistics are reported in the results section. The wide-field visual projection set-up did not allow for eye-tracking. However, to promote stable fixation during all conditions, the fixation point was continuously visible at a fixed position on the screen, and only expert subjects with a good fixation stability were used. In all experiments, fixation distance and head alignment were held constant by a chin rest mounted inside the head coil. Subjects’ heads were stabilized with foam padding to minimize movement during the scans.

### Imaging parameters

Single-shot echo-planar imaging (EPI) images were acquired with interleaved slice ordering using blood-oxygenation-level-dependent imaging^79^. For all fMRI experiments we acquired thirty 2.5 mm thick slices (no gap) perpendicular to the calcarine sulcus (in-plane resolution 3 × 3 mm; repetition time (TR) 2 s; echo time (TE) 30 ms, flip angle 70 deg; bandwidth 2298 Hz/pixel; field of view (FOV) 192 × 192 mm). The number of volumes per acquisition scan was 156 for the crowding experiment, 128 for the localizer scans, and 256 for the retinotopic mapping experiment. In each scan, the first four volumes were discarded from data analysis in order to achieve a steady state, and the stimuli started at the beginning of the fifth volume.

Each participant also underwent three structural scans for cortical surface reconstruction (T1-weighted sagittal Magnetization Prepared Rapid Gradient Echo (MPRAGE) sequence, TR = 2 s, TE = 4.38 ms, TI = 910 ms, flip angle = 8 deg, 1 mm^3^ voxels, matrix 256 × 256, 176 contiguous slices, bandwidth = 130 Hz/pixel).

### Data analysis

Behavioral data collected inside the scanner was analyzed with a group-level ANOVA treating subjects as a random factor and included two within-subjects factors: Orientation Difference (2 levels: similar (strongly crowded) and dissimilar (weakly crowded) and Stimulus type (3 levels: all-the-same, one-different, and all-different). Post-hoc paired *t*-tests were Bonferroni corrected. Cohen’s *d* for correlated measures was used to describe the standardized mean difference of the crowding effect

$${\rm{Coehn}}\mbox{'}{\rm{s}}\,{d}_{z}=\frac{{{\rm{M}}}_{{\rm{diff}}}}{\sqrt{\frac{\sum {({{\rm{X}}}_{{\rm{diff}}}-{{\rm{M}}}_{{\rm{diff}}})}^{2}}{N-1}}}$$

where the numerator is the difference between the mean (M) of the difference scores and the comparison value μ, and the denominator is the standard deviation of the difference scores (S_diff_)^80^.

The analysis of structural and of retinotopic data was performed using UCSD/UCL FreeSurfer^81^ based on standard procedures described in detail elsewhere^36,37,77,78^. Briefly, the three high-resolution structural images, obtained from each subject, were manually registered and averaged. The skull was stripped off by expanding a stiff deformable template out to the dura, the gray/white matter boundary was estimated with a region-growing method, and the result was tessellated to generate a surface that was refined against the MRI data with a deformable template algorithm. By choosing a surface near the gray/white matter border (rather than near the pial surface, where the macrovascular artifact is maximal), we were able to assign activations more accurately to the correct bank of a sulcus. The surface was then unfolded by reducing curvature while minimizing distortion in all other local metric properties. After reconstruction, each hemisphere was then completely flattened using five relaxation cuts: one cut along the calcarine fissure, three equally spaced radial cuts on the medial surface, and one sagittal cut around the temporal lobe.

Functional data from retinotopic mapping scans underwent several pre-processing steps including motion correction, averaging across the two scans for each stimulus type (eccentricity and polar angle) in order increase the signal to noise ratio, and aligning to the structural images. Phase-encoded retinotopic data were then analyzed by voxelwise Fourier transforming the fMRI time series (after removing constant and linear terms). This Fourier analysis generates real and imaginary components (equivalently, amplitude and phase) at each frequency. To estimate the significance of the BOLD signal modulation at the stimulus frequency (eight cycles per scan), the squared Fourier amplitude was divided by the summed mean squared amplitude (power) at all other frequencies, which includes noise. The second harmonic of the stimulus frequency and very low frequencies (1 and 2 cycles per scan, residual motion artifacts) were ignored. Response phase at the stimulus frequency was used to map retinotopic coordinates (polar angle or eccentricity). Boundaries of retinotopic cortical areas were defined on the cortical surface for each subject based on the visual field sign analysis. The field sign analysis provides an objective means of drawing borders between areas based on the angle between the gradients (directions of fastest rate of change) in the polar angle and eccentricity with respect to the cortical surface^36^.

Functional images from the main experiment and localizer scans were pre-processed and analyzed using SPM12 (Wellcome Department of Cognitive Neurology). Pre-processing steps included correction for head motion and for slice acquisition timing using the middle slice as a reference, and aligning to the structural images. Images were analyzed in native space in order to be mapped onto the corresponding cortical surface reconstruction. A small amount of smoothing (3 mm FWHM) was applied to localizer data only. The effect of each stimulus type (for the localizer scans) or of the combination of Attention and Orientation Difference (for the crowding experiment) was estimated on a voxel-by-voxel basis, according to the general linear model (GLM) as implemented in SPM. Blocks in the localizer scans were modeled as box-car functions, and trials in the crowding experiment were modeled as gamma functions. Both were convolved with a canonical hemodynamic response function. For the crowding experiments, two different GLMs were estimated that modeled the series of stimuli which were simultaneously presented to the left and to the right hemifield, respectively. Each hemifield-specific GLM was used to analyze the data from the contralateral hemisphere, under the assumption that early visual regions exhibit almost exclusive contralateral activation. Note that while this procedure does not exclude from the analysis the contribution of top-down feed-backs and interhemispheric interactions from homologous areas, it is not ideal to isolate the contribution of the ipsilateral activity.

We first used retinotopic mapping to define the borders between striate and extrastriate visual areas. We then selected for each participant the eight visual regions representing the lower visual field, where the stimuli array in the crowding experiment were located, i.e., V1d, V2d, V3, V3A, V7, V6, V6Av, and V4/V8. These visual regions were successfully defined in all the 12 subjects which performed the retinotopic experiment (24/24 hemispheres). We excluded from the analysis those lower-field regions that we were not able to map in all subjects (as the LOR region that was also activated in a minority of cases).

The localizer scan was then used to define the portion of each lower-field visual region including the target presented in the crowding experiment. For each participant, we computed a statistical parametric map of the contrast between random dynamic white noise vs. the fixation baseline based on the localizer scan data. The map was thresholded at p < 0.001, corrected for multiple comparisons using false discovery rate (FDR), projected on the participant’s cortical surface, and intersected with the dorsal representation of the retinotopic regions to define a set of regions of interest (ROIs) that were significantly activated by the dynamic white-noise stimulus. The rationale behind this approach was to consider, for each retinotopic region, the portion of the cortex centered on the target. However, because of the properties of the BOLD signal and the magnet field, the actual size of this window, when projected into the cortex, corresponds to a bigger area which includes both target and presumably some part of the flankers. The center-to-center distance between the two flankers subtending the target was about 3 deg of angle in the circumferential direction. This distance, at 8 deg of eccentricity, corresponds roughly to 3 mm of cortex in V1^82^. At 3 Tesla, the spatial point spread function (PSF) of the fMRI BOLD signal has an FWHM of about 3.5 mm^83^. We smoothed the data by a Gaussian kernel with FWHM of 3 mm, increasing the PSF to about 4.5 mm. A circle with the diameter of 4.5 mm on V1, when projected back to the visual space, has a diameter of about 4.5 deg, so above the size of the target. To exclude that the results might depend on the ROI size, so on the inclusion of some parts of the flankers or non-stimulated cortex, we included a control analysis on the unmasked retinotopic regions.

The resulting masked ROIs were well localized but consequently also very small. In several cases, we were not able to define all ROIs because there was no significant activation in the localizer scan within each retinotopic region.

The localizer experiment was also used to create a set of VWFA ROIs, defined in every participant as a contiguous cluster of voxels close to V4/V8 in the left fusiform gyrus, from the contrast Latin vs. Devanagari characters (p < 0.001, FDR corrected).

For the crowding experiment, from each of the two hemifield-specific GLMs, we took parameter estimate images for each condition (i.e. each combination of Attention and Orientation), which represent percent signal change in each voxel relative to the fixation baseline. We then projected the images to the participant’s cortical surface and averaged them across all vertices within each of the participant-specific ROIs belonging to the contralateral hemisphere. This resulted, for each participant, in an estimate of activation induced in each retinotopically-constrained region by stimuli presented in the contralateral hemifield under the various conditions. A similar procedure was employed for the VWFA. We then conducted a repeated-measures ANOVAs on these regional activation estimates, with factors Attention (attended vs. unattended) and Orientation (similar vs. dissimilar).
